# Enzymatic Degradation of *Cryptosporidium* spp. Oocysts: A Combined In Silico and In Vitro Study

**DOI:** 10.3390/molecules31162919

**Published:** 2026-08-21

**Authors:** Débora Castro Toledo de Souza, Ana Carolina Silva, Adriane Toledo Batista da Silva, Ruth Celestina Condori Mamani, Júlia Gomes de Carvalho Jorge, Carolina Magri Ferraz, Fábio Ribeiro Braga, Jackson Victor de Araújo, Filippe Elias de Freitas Soares

**Affiliations:** 1 Laboratory of Biotechnology and Applied Biochemistry, Department of Chemistry, Federal University of Lavras, Lavras 37200-900, MG, Brazil; contatodeboracsouza@gmail.com (D.C.T.d.S.); silva.a.c5444@gmail.com (A.C.S.); adriane.silva5@estudante.ufla.br (A.T.B.d.S.); ruthcondori20@gmail.com (R.C.C.M.); 2 Department of Veterinary Clinics and Surgery, School of Veterinary Medicine, Federal University of Minas Gerais, Belo Horizonte 31270-901, MG, Brazil; zooagrobrasil@gmail.com; 3 Laboratory of Experimental Parasitology and Biological Control, University Vila Velha, Vila Velha 29102-920, ES, Brazil; carolina.ferraz@uvv.br (C.M.F.); fabio.braga@uvv.br (F.R.B.); 4 Department of Veterinary Medicine, Federal University of Viçosa, Viçosa 36570-900, MG, Brazil; jvictor@ufv.br

**Keywords:** proteases, biochemical control, zoonoses, one health

## Abstract

Proteases are widely studied hydrolases as “green technologies” for degrading structures within complex matrices, although combining different classes poses challenges for catalytic stability. To the best of our knowledge, this is the first study to specifically evaluate the biochemical control of *Cryptosporidium* spp. oocysts using plant- and microbial-derived proteases. This study evaluated the compatibility and sanitizing potential of the microbial serine protease subtilisin Carlsberg (HPF-1SCA) and the plant cysteine protease papain (1PPP), both individually and in a 15% (*w*/*v*) combination, hypothesizing a synergistic effect due to their distinct catalytic specificities, as a potential biochemical approach to reduce contamination by these zoonotic protozoa of global importance, which are highly resilient to conventional disinfectants. The experimental design comprised five distinct treatment groups: a negative control (distilled water), a positive chemical control (0.04% *v*/*v* NaClO), treatment with isolated papain (15% *w*/*v*), treatment with isolated microbial HPF formulation (15% *w*/*v*), and a combined treatment using both enzymes simultaneously (15% *w*/*v* each). Concurrently, an in silico molecular docking investigation was conducted to elucidate the predicted binding scores, structural compatibility, and preferential binding mechanisms of these enzymes toward the Cryptosporidium Oocyst Wall Protein (COWP). In vitro compatibility assays revealed an immediate antagonistic effect due to mutual proteolysis, reducing overall proteolytic activity by 45% after 72 h. Despite this antagonism, the enzyme mixture (G5) and the isolated microbial protease (G4) achieved a 93% reduction in oocysts, equivalent to the conventional 0.04% NaClO treatment (G2), while papain (G3) achieved 65%. All these results represented statistically significant efficacy (*p* < 0.01) compared to the negative control (G1). In silico molecular docking studies provided a structural predictive basis for the experimental data, suggesting that subtilisin Carlsberg (HPF-1SCA) exhibits more favorable predicted binding scores and structurally compatible interfaces, compared to papain, particularly toward the major oocyst structural proteins COWP8 (estimated ΔG = −15.2 kcal·mol^−1^) and COWP6 (estimated ΔG = −13.3 kcal·mol^−1^). This provides robust evidence for initial target recognition and molecular anchoring, although these static models do not definitively confirm catalytically productive cleavage conformations. In summary, this in vitro proof-of-concept demonstrates that microbial proteases show promise for the biochemical destabilization of oocysts without generating toxic chlorinated byproducts. While these baseline findings align with the One Health concept, future field-scale validations assessing enzyme stability under variable environmental conditions and in vivo infectivity assays are required before practical application in sanitation protocols.

## 1. Introduction

Enzymes are essential biocatalysts that facilitate chemical reactions vital to life with high specificity and efficiency [1]. They are classified according to their catalytic mechanisms and the amino acid residues located within their active sites [2]. Among these, hydrolases are enzymes whose primary role is to catalyze the hydrolysis of peptide bonds in proteins and peptides; they are categorized under the Enzyme Commission code EC 3.4 [3]. The subclass of these enzymes is directly associated with the amino acid serving as the primary nucleophile in the active site, notably including cysteine proteases (EC 3.4.22—of plant origin, such as papain) [4,5] and serine proteases (EC 3.4.21—of microbial origin, such as subtilisins) [6,7].

The function of these macromolecules depends on their catalytic cleft, a specific three-dimensional region where the substrate binds and undergoes nucleophilic attack, resulting in cleavage of the polypeptide chain [8]. Furthermore, due to their efficient and clean mechanism of action, proteases feature in biotechnology research as a “green technology” [9]. Their ability to hydrolyze structures vital to the life cycle of various pathogens represents an innovative biochemical control tool capable of replacing reliance on chemical disinfectants that are harmful to health [10]. However, developing efficient biotechnological formulations poses the challenge of evaluating compatibility and catalytic stability with respect to the specific substrates of interest [1,3].

The use of proteases opens an innovative frontier in biosafety and environmental hygiene, offering a promising avenue for research into controlling highly resilient organisms such as *Cryptosporidium* spp. oocysts [11]. Cryptosporidiosis is not merely a clinical gastrointestinal infection; it is a complex public health issue and a persistent challenge for global health surveillance [12]. This complexity stems from the parasite’s resilient biology, its zoonotic nature, which significantly affects both humans and livestock, and the severe limitations of current therapies and disinfection methods [11,13,14].

*Cryptosporidium* spp. comprises at least 30 validated species. Among them, *Cryptosporidium parvum* stands out for its significant impact on dairy herds, where neonatal calves serve as the primary epidemiological reservoir for human infections [15,16]. Its monoxenous life cycle culminates in the shedding of sporulated oocysts in feces, which are transmitted via the fecal-oral route through ingestion of contaminated water or food. Clinically, the disease causes fever, vomiting, and severe diarrhea, and is a leading cause of morbidity and infant mortality from dehydration in developing countries [17,18]. In developed regions, outbreaks among immunocompetent populations are frequently linked to contamination of water supply systems, whereas chronic and fatal cases affect immunocompromised individuals [19,20].

One of the major challenges in sanitary surveillance is the extreme resistance of *Cryptosporidium* spp. oocysts to conventional water disinfection methods, particularly chlorination [21]. This structural resilience necessitates high chemical doses or prolonged exposure times, alternatives that are economically unfeasible and operationally hazardous because of the risk of generating toxic chlorinated by-products [22]. To overcome this biological barrier without harming the ecosystem, sanitary strategies must focus on destabilizing the outer shell and, crucially, dismantling the intricate network of structural proteins (cysteine-rich glycoproteins) that imparts rigidity to the oocyst wall [23].

Structurally, *C. parvum* oocysts have a rigid outer bilayer composed of lipids and glycoproteins, while the internal structure is characterized by the presence of proteins rich in cysteine (Cys), histidine (His), and tyrosine (Tyr) [24,25,26]. This unique amino acid composition is presented here as a biological rationale for selecting these receptors as key structural targets—since their extensive cross-linking is fundamental to oocyst resistance—rather than as a predictive map for linear cleavage sites [23]. Proteomics studies and high-resolution microscopy have shown that the *C. parvum* oocyst wall is composed primarily of the COWP (Cryptosporidium Oocyst Wall Protein) family, which accounts for approximately 75% of the structural matrix [27]. Although COWP1 is the most abundant (55%), proteins such as COWP8 (13%) and COWP6 (8%) constitute a highly significant portion of this matrix and share the exact same dense cysteine-rich architecture capable of forming extensive networks of disulfide bridges, which are responsible for the shell’s rigidity and impermeability. In addition, *Cryptosporidium parvum* Surface Protein 1 (CpSP1), while not belonging to the classical COWP family, is strategically located on the outer surface of the oocyst, representing a potential initial point of contact for enzymatic degradation [28]. These findings reinforce that the overall integrity of the wall depends on the synergistic network of COWPs. Therefore, targeting these key matrix components (COWP6 and COWP8) alongside surface proteins (CpSP1) provides a robust and highly representative model for evaluating the biochemical destabilization of the parasite’s protective barrier.

To overcome these limitations and evaluate target binding potential without relying solely on empirical benchtop assays, modern biochemical engineering relies heavily on computational structural biology [29,30]. Molecular docking, for example, targets catalytic clefts and maps three-dimensional interactions at the atomic scale [31]. This approach estimates the binding affinity (predicted ΔG) and the dissociation constant (Kd), clarifying that these measures provide a structural basis for assessing the binding likelihood and interfacial compatibility of enzyme–receptor complexes, as well as determining whether active sites recognize structurally conserved motifs across different protein receptors [24,32].

Given this scenario, the search for clean, sustainable technologies that reduce reliance on persistent chemical agents has become imperative, aligning with the development goals of the UN 2030 Agenda [33]. Although the catalytic activity of proteases in digesting nematode and arthropod cuticles is well documented in the literature [2,4,34], directing these hydrolases to cleave the protein matrices that protect protozoan oocysts establishes a promising new paradigm.

Therefore, the main hypothesis of this study is that proteases can catalytically degrade the highly cross-linked protein matrix of the oocyst wall, compromising its structural integrity. The scientific rationale for combining a plant cysteine protease (papain) with a microbial serine protease (subtilisin) was to explore a potential synergistic effect, with the expectation that their distinct catalytic specificities would cleave a broader spectrum of peptide bonds in the complex matrix, thus exhibiting superior performance compared to individual enzymes. Therefore, the present study examined the capabilities—both individual and combined—of proteases as a strategy for the in vitro biochemical regulation of *Cryptosporidium* spp. oocysts, complemented by in silico molecular docking, to evaluate the predicted binding affinity, thermodynamic affinity, and structural interactions of these enzymes with oocyst wall proteins.

## 2. Results

### 2.1. Enzyme Compatibility and Stability Tests

**Figure 1:**
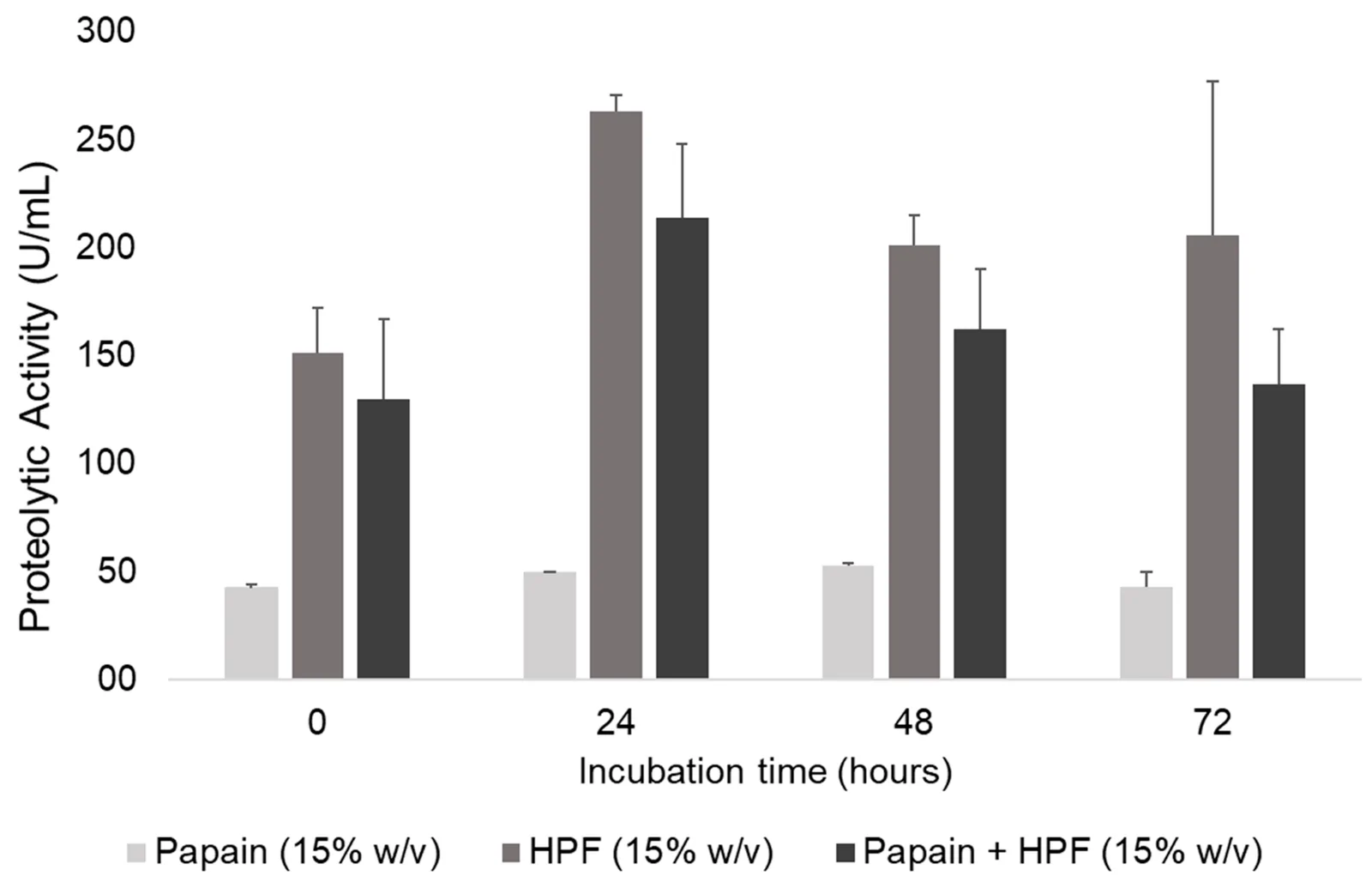
Stability and enzymatic compatibility profile of isolated and combined proteases over time. (G1) *Bacillus licheniformis* microbial protease (HPF) at 15% (*w*/*v*); (G2) Papain at 15% (*w*/*v*); and (G3) Equal combination of HPF and papain (15% *w*/*v* each). Values are expressed as enzymatic activity per milliliter (U/mL) ± standard deviation (SD) of *n* = 3 independent replicates. Statistical significance between the combined formulation and the isolated microbial protease was confirmed using Student’s *t*-test (*p* < 0.05). One unit of activity (U) is defined as the amount of enzyme required to release 1 µg of tyrosine per minute under the standardized conditions of the caseinolytic assay.

Enzyme compatibility tests showed that combining the two proteases (papain and the commercial HPF formulation, whose major component is subtilisin Carlsberg) resulted in immediate inhibition. Statistically, the proteolytic activity of the combination did not differ significantly from that of the HPF formulation alone, whereas both treatments exhibited significantly higher activity than papain alone (*p* < 0.01). When papain was added to the HPF solution, the mixture’s proteolytic activity was lower than the theoretical sum of the individual activities. Immediately after mixing (0 h), a 33% reduction in proteolytic activity was observed relative to the sum of the individual activities. Statistical inference using Student’s *t*-test confirmed that the proteolytic activity of the combined formulation was significantly lower (*p* < 0.05) than the theoretically expected sum and the activity of the isolated microbial protease across all evaluated time points. This trend of catalytic activity loss remained stable at 32% after 24 h of incubation, rose slightly to 36% after 48 h, and reached a peak reduction of 45% after 72 h of incubation (Figure 1).

### 2.2. In Vitro Efficacy and Cytotoxic Effect on Cryptosporidium spp. Oocysts

Results from in vitro assays on *Cryptosporidium* spp. oocysts showed that conventional chemical treatment with 0.04% (*v*/*v*) NaClO in the positive control group (G2) achieved the highest efficacy, with 93% mortality. Notably, the groups treated with the isolated microbial enzyme HPF (G4) and with the combined action of HPF and papain (G5) both exhibited 93% mortality. There was no statistically significant difference (*p* > 0.01) between the two enzymatic treatments (G4 and G5) and the positive control (G2), highlighting the sanitizing potential of the hydrolases.

**Figure 2:**
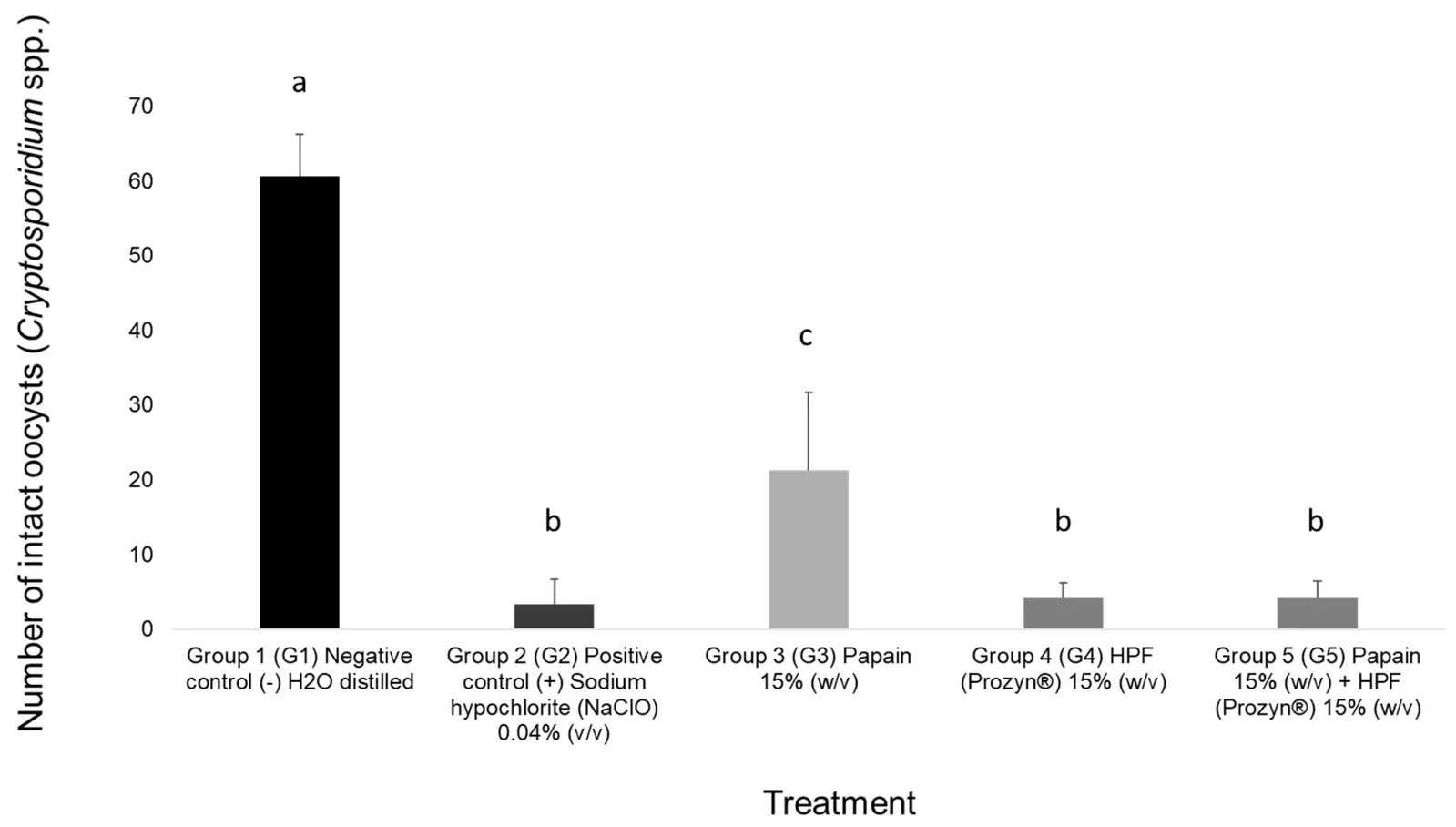
Number of intact *Cryptosporidium* spp. oocysts was counted after incubation of the groups at 26 ± 1 °C in the dark for 72 h. The groups received the following treatments: Group 1 (G1) Negative control with water (distilled H_2_O); Group 2 (G2) Positive control with 0.04% (*v*/*v*) sodium hypochlorite (NaClO); Group 3 (G3) Papain at 15% (*w*/*v*); Group 4 (G4) Microbial enzyme—HPF formulation (Prozyn^®^—Brazil, São Paulo, Brazil) at 15% (*w*/*v*); Group 5 (G5) Combination of the papain and the microbial enzyme—HPF formulation (Prozyn^®^—Brazil), both at 15% (*w*/*v*). Data represent the mean ± SD of *n* = 6 independent biological replicates per group. Statistical comparisons were performed using One-Way ANOVA followed by Tukey’s HSD post-hoc test. Groups G2, G4, and G5 showed no statistically significant differences among themselves (*p* > 0.05), differing only from G1 and G3 (*p* < 0.01).

In contrast, the group treated exclusively with the plant enzyme papain (G3) showed moderate efficacy, with 65% mortality; this result was statistically inferior to and distinct from those of the other treatment groups (G2, G4, and G5). All groups treated with sanitizing agents (G2 through G5) differed significantly (*p* < 0.01) from the negative control (G1, treated only with distilled H_2_O), in which the parasites’ cellular integrity was preserved (Figure 2).

**Figure 3:**
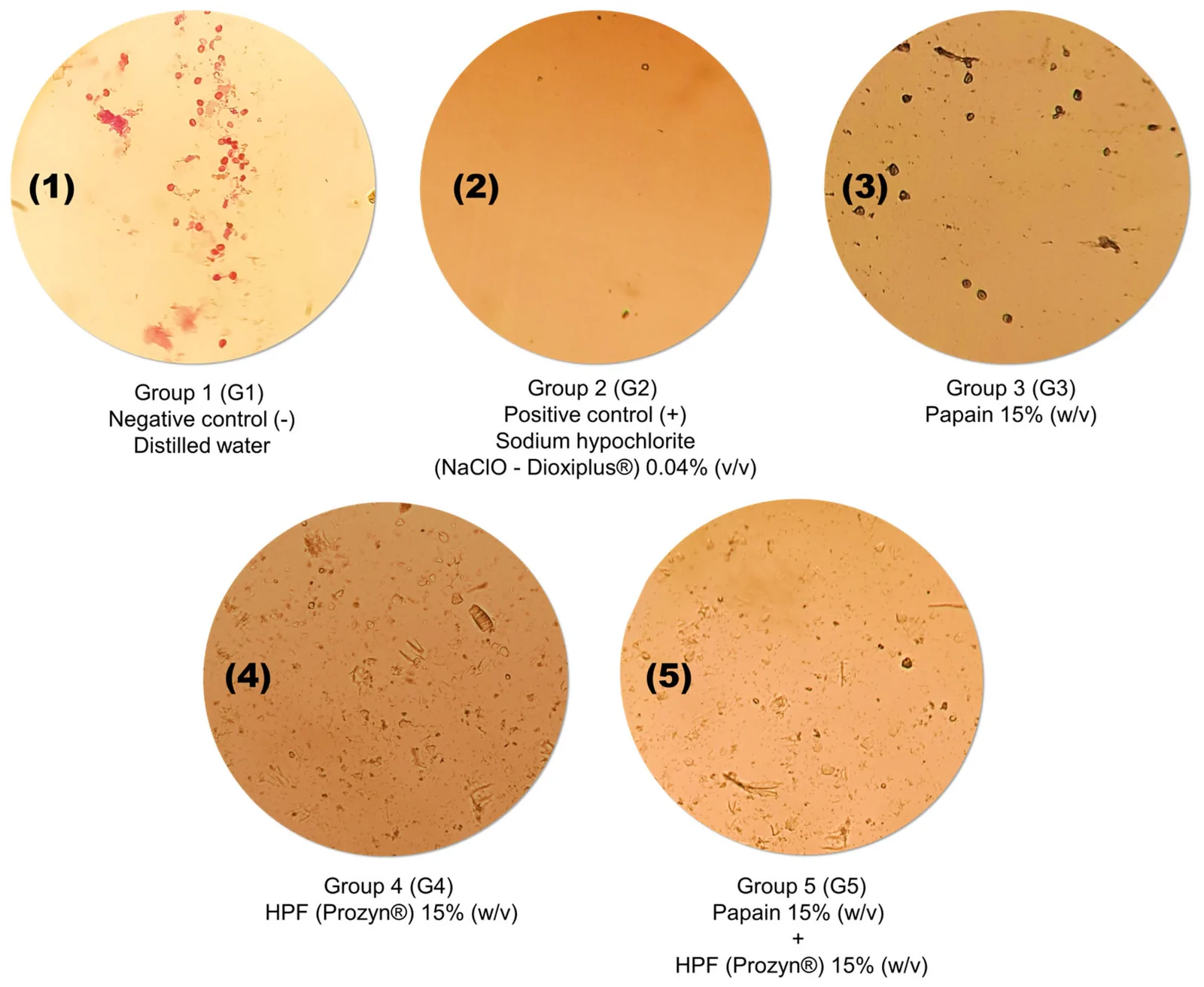
Microphotographs of *Cryptosporidium* spp. oocysts subjected to different experimental treatments. Initial Suspension: High density of intact oocysts prior to exposure to the treatments. Group 1 (G1) Negative control with water (distilled H_2_O). Group 2 (G2) Dioxiplus^®^ positive control—(0.04% *v*/*v* sodium hypochlorite), demonstrating that the numerical density was maintained, but with changes in viability. (G3 and G4) Groups treated with the microbial enzyme HPF Prozyn^®^ and in combination with 15% papain (*m*/*v*), respectively, showing a significant reduction in the count of visible oocysts. Group 5 (G5) Combination of the papain and the microbial enzyme—HPF formulation (Prozyn^®^—Brazil), both at 15% (*w*/*v*). (G2) Group treated with 15% papain (*m*/*v*), exhibiting a moderate reduction in parasite concentration. Images captured using a high-resolution digital imaging system attached to the eyepiece of an optical microscope (400× magnification) following modified Ziehl-Neelsen staining. (Scale bar = 2 µm).

Hydrolytic and physical degradation resulting from catalytic activity was confirmed by direct microscopic analysis. Oocysts exposed to the enzymatic formulations exhibited severe structural collapse, fragmentation of the outer shell, and a drastic reduction in the density of intact parasitic bodies in the suspension, effects that mimicked the action of the chemical control and contrasted with the morphological integrity observed in the negative control group (Figure 3).

### 2.3. In Silico Molecular Docking Analysis (Protein-Protein)

Table 1 and Table 2 present a summary of the selected docking models between oocyst wall proteins (*Cryptosporidium* spp.)—COWP6, COWP8, and CpSP1—and the proteases papain (Table 1)—1PPP—and Carlsberg subtilisin (Table 2)—1SCA—the enzyme used in the commercial HPF formulation.

**Figure 4:**
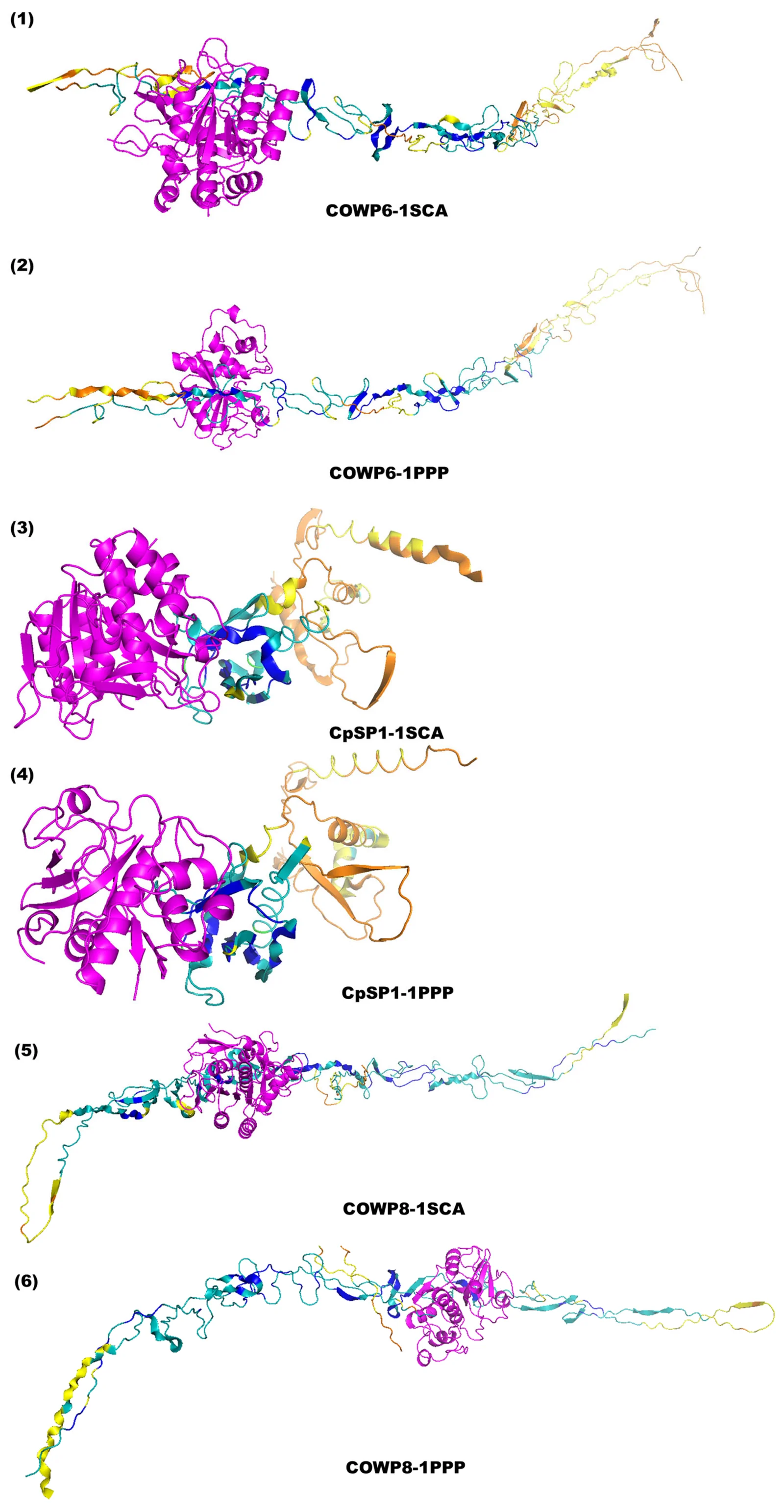
Selected protein–protein docking models between the proteases papain (1PPP) and Carlsberg subtilisin (1SCA) and the structural proteins of the *Cryptosporidium* spp. oocyst wall (COWP6, CpSP1, and COWP8). The specific complexes are displayed as follows: (1) COWP6-1SCA; (2) COWP6-1PPP; (3) CpSP1-1SCA; (4) CpSP1-1PPP; (5) COWP8-1SCA; and (6) COWP8-1PPP. The enzyme chains are shown in a fixed contrasting color (magenta), as they are experimentally determined structures. The predicted COWP receptors are colored according to the standard AlphaFold pLDDT confidence scheme: very high confidence (pLDDT > 90, blue), confident (90 > pLDDT > 70, teal), low confidence (70 > pLDDT > 50, yellow), and very low confidence (pLDDT < 50, orange). This graphical representation allows for a direct comparison of the predicted interaction profiles while accurately reflecting the regional structural reliability of the AF2 models.

For each receptor, the best interaction models were identified based on the values of free energy of binding (ΔG), dissociation constant (Kd), and intermolecular contact (IC) profiles. This consolidation allowed us to map the predicted interactions at the contact interface, highlighting the COWP matrix proteins as the primary targets for stable anchoring and the surface protein CpSP1 as the component with the least favorable predicted binding scores to proteolytic attack. Following numerical screening, the predicted three-dimensional contact interfaces, and electrostatic interactions established at the active site of each protease were graphically represented, focusing strictly on spatial fit and structural reliability without depicting speculative residue-level polar interactions (Figure 4).

To transition from a descriptive analysis to testable biochemical hypotheses, a comprehensive residue-by-residue identification of the predicted binding interfaces for all complexes was performed (Supplementary Table S1). A focused side-by-side comparison between the most favorable (COWP8-1SCA) and least favorable (CpSP1−1SCA) predicted interactions clarifies how structural features relate to their thermodynamic differences. In the highly favorable COWP8-1SCA complex (estimated ΔG = −15.2 kcal/mol), the predicted interface is extensive because it relies on a broad network of interactions involving several structural residues (e.g., Met-142, Leu-155, and Ser-160), ensuring strong molecular recognition and stable anchoring. Conversely, the less favorable CpSP1-1SCA complex (estimated ΔG = −12.7 kcal/mol) relies on a significantly smaller, shallower interface, which is consistent with lower structural compatibility. Notably, our rigid-body docking models captured highly stable anchoring poses rather than exact transition-state catalytic conformations, so direct contacts between the catalytic triad and potential cleavage targets (cysteines or tyrosines) were generally not observed within the 4.0 Å threshold. This structural difference supports COWP8 as a highly susceptible target for enzyme anchoring and provides a mechanistic rationale for the robust degradative efficacy observed in vitro, while establishing testable hypotheses for future dynamic structural studies.

## 3. Discussion

This research, the mean data for each group suggest that the protease-mediated in vitro reduction potential is as effective as that of the conventional positive control (Figure 3). This finding is significant because it demonstrates the potential of this protease-mediated biochemical control. These results support the feasibility of further research into this technology, given its relevance to disease control within the One Health framework.

Moreover, enzyme compatibility studies revealed an immediate (0 h) antagonistic effect upon binding of the two proteases, resulting in a 33% loss of total proteolytic activity, which increased to 45% at 72 h. This phenomenon suggests competitive inhibition or, more likely, mutual proteolysis, in which one enzyme degrades the other enzyme’s peptide structure due to a lack of absolute substrate specificity. In light of this, when correlating the antagonistic effect with the in vitro experiments, a notable kinetic behavior was observed. Despite the substantial reduction in the mixture’s overall enzymatic activity, the group treated with the combined action (G5) maintained 93% oocyst mortality, a value statistically equivalent to the treatment with HPF alone (G4—93%) and the positive control (G2—95%).

This scenario suggests that either the residual proteolytic activity of the mixture or the initial and immediate action of the HPF enzyme—before it was severely inhibited by papain—was sufficient to irreversibly compromise the integrity of the cell wall of *Cryptosporidium* spp. oocysts. From the perspective of environmental hygiene and operational safety, this finding is noteworthy, given that high concentrations of chlorine can lead to the formation of toxic byproducts, such as trihalomethanes (THMs) and chlorinated hydrocarbons, which can have adverse ecological impacts and pose risks to One Health [35]. Similarly, conventional disinfectants such as glutaraldehyde require prolonged immersion times and can cause corrosion of surfaces and equipment [13,36]—limitations that could be overcome by using purified hydrolases. Therefore, although co-administration compromises the long-term biochemical stability of the solution, it does not negate the sanitizing potential during the first few hours of contact, validating the robustness of microbial protease in environmental hygiene scenarios.

In the present work, structural damage was observed in oocysts subjected to enzymatic treatment, an effect not observed in the initial suspension consisting solely of distilled water. Since proteases catalyze the hydrolysis of peptide bonds, we hypothesize that this damage results from the catalytic degradation of the oocyst’s structural proteins [37,38]. However, while optical microscopy provides visual confirmation of this structural collapse, direct biochemical validation is necessary to prove the exact mechanisms of enzymatic destruction definitively.

Proteomics studies and high-resolution microscopy have shown that the *C. parvum* oocyst wall is composed primarily of the COWP family, which accounts for approximately 75% of the structural matrix. Among these, COWP1 is the most abundant (55%), followed by COWP8 (13%) and COWP6 (8%), all of which are rich in cysteine residues capable of forming extensive networks of disulfide bridges, which are responsible for the shell’s rigidity and impermeability [23,24]. CpSP1, in turn, does not belong to the classical COWP family but is located on the outer surface of the oocyst and is considered a promising diagnostic target, although it is less relevant to structural degradation [39]. These findings reinforce that the integrity of the wall depends primarily on the dense matrix formed by the COWPs, and that enzymatic action on these proteins is critical for compromising the parasite’s viability.

The in silico results provide supportive structural rationale for the trends obtained in vitro, suggesting that these enzymes possess complementary binding interfaces for key structural components of the oocyst wall, thereby providing a structural predictive basis for screening. Therefore, our computational approach specifically evaluated three-dimensional spatial fit, active site accessibility, and predicted binding affinity (estimated ΔG) through protein-protein docking, rather than performing a sequence-based cleavage motif mapping [32]. Overall, the binding assays indicated that Carlsberg subtilisin (1SCA, EC 3.4.21.62), the core enzyme of the HPF microbial blend, exhibits more favorable predicted binding scores compared to papain (1PPP, EC 3.4.22. 2), forming complexes with more negative estimated ΔG values and lower estimated dissociation constants (Kd, mol L^−1^) for all structural proteins evaluated.

In the generated models, papain exhibited more reliable interactions, particularly with the structural protein COWP8; however, its relatively lower affinity explains the reduced efficacy observed in vitro. Furthermore, the genetic study by Bacchetti et al. [23] demonstrated that deletion of the COWP8 protein is not sufficient to alter the mechanical strength or even the structural resistance of the oocyst, which biologically justifies papain’s limited impact when targeting this site.

On the other hand, subtilisin resulted in models with highly favorable predicted binding scores, especially with the remaining major structural proteins of the oocyst wall matrix, COWP8 (estimated ΔG = −15.2 kcal/mol and Kd = 6.9 × 10^−12^ M) and COWP6 (estimated ΔG = −13.3 kcal/mol and Kd = 1.7 × 10^−10^ M). Together, these two proteins account for approximately 21% of the structural matrix and share the dense, cysteine-rich architecture responsible for maintaining wall rigidity [14]. The strong predicted affinity of subtilisin for these key matrix targets provides a robust structural hypothesis for its superior degradative efficacy (>90%) observed in the in vitro assays.

Analysis of the predicted intermolecular contact (IC) profile revealed that the favorable binding scores between the subtilisin binding site and the structural proteins are predicted to be driven by the formation of an extensive network of nonpolar-nonpolar interactions (hydrophobic effect) associated with efficient networks of charged-polar and polar-polar contacts (hydrogen bonds) at the active site interface [31,39]. Importantly, the structural evaluation confirmed that the enzyme’s binding interface actively overlaps with its catalytic cleft, ensuring that the highest-ranked docking poses position the structurally ordered regions of the COWPs (confident domains, pLDDT > 70) directly into the proteases’ active sites. Regions modeled with low confidence (70 > pLDDT > 50) and very low confidence (pLDDT < 50) were not considered primary contributors to the evaluated binding affinity, as their intrinsic disorder in monomeric prediction likely misrepresents the heavily cross-linked, rigid state of these cysteine-rich proteins in the native oocyst wall matrix [23]. This profile of favorable predicted inter-residue contacts explains the high structural compatibility of the enzyme complex obtained in the computational model and supports the biological potential observed in in vitro treatment with the microbial enzyme.

In this context, although CpSP1 is presented by Wang et al. [39] as a relevant target protein due to its surface location, it exhibited smaller and less structurally compatible interfaces in the generated models, suggesting lower susceptibility to proteolytic degradation by these enzymes. These in silico findings support the hypothesis that highly favorable predicted binding scores toward COWPs are a key structural prerequisite for potential wall destabilization. Building upon this residue-level analysis, a transparent assessment of the mechanistic relevance of these computational models is essential. While the in silico approach successfully identifies thermodynamically favorable interfaces, it must be explicitly acknowledged that rigid-body docking algorithms inherently represent static approximations. Consequently, some of the predicted complexes may represent transient, non-productive binding events rather than catalytically relevant poses. This occurs because enzymatic hydrolysis by proteases requires highly specific, sub-angstrom geometric alignment between the enzyme’s catalytic triad and the target scissile peptide bond—a dynamic transition-state process that static rigid docking cannot fully capture [2,4]. Therefore, if certain highly ranked poses do not exhibit a perfect geometric overlap with the target cleavage sites (such as specific cysteines and tyrosines), this constitutes a strict limitation of the static models. In this context, our structural predictions should be carefully interpreted as robust evidence of molecular recognition and structural susceptibility of the oocyst wall matrix, which strongly supports the degradative efficacy observed in vitro. However, these models do not definitively confirm catalytically productive configurations, which would ultimately require validation through time-resolved structural biology or advanced kinetic mutagenesis studies [35].

The literature indicates that eradicating *Cryptosporidium* spp. is challenging due to the high resilience of oocysts to conventional disinfectants, as well as their structural robustness and microscopic size [18]. Effective control requires both the management of infected hosts and the environmental inactivation of oocysts. The complexity of mitigating environmental dispersal also lies in the size of the oocyst (4 to 6 μm in diameter), which allows them to evade multiple barriers and conventional municipal water filtration strategies, thereby perpetuating water contamination [40]. Although drugs exist for the treatment of cryptosporidiosis, none are fully effective in terms of clinical and parasitological responses [18].

While the results show a significant in vitro reduction in oocysts, this proof-of-concept study has key limitations. The 15% (*w*/*v*) enzyme concentration is high, and non-specific mechanisms like osmotic stress or protein denaturation may contribute to damage, not just targeted cleavage. Such high enzyme levels are not feasible for large-scale water sanitation, although other protocols like hydrogen peroxide use similarly high concentrations. This approach is best suited as a complementary strategy for niches like veterinary or agricultural pretreatments, not as a universal disinfectant. Future work must identify the minimum effective dose through dose–response testing. The use of a proprietary microbial blend (HPF) complicates attribution, requiring validation with purified subtilisin. The oocysts were from naturally infected calves without species identification, though *C. parvum* is likely the main agent. The in silico targets were based on *C. parvum* COWPs, which are well characterized and provide a theoretical basis for the in vitro results, but direct biochemical proof is still needed.

To date, no studies have been identified that specifically evaluate the biochemical control of *Cryptosporidium* spp. oocysts by plant- and microbial-derived proteases. However, Siqueira et al. [38] investigated *C. papaya* latex and the enzyme papain as a sustainable strategy against bovine coccidiosis. In vitro assays demonstrated that 30% (*m*/*v*) enzymatic solutions catalyzed degradation of the outer membrane of *Eimeria bovis* oocysts, causing the parasite’s internal contents to leak out. In this context, papain, a plant protease extracted from *C. papaya*, has been recognized for its anthelmintic activity in the classical literature since the 1940s [41].

Meanwhile, enzymes of microbial origin have been studied to maximize their industrial and health applications. In this regard, the species *B. licheniformis* is widely recognized for its biotechnological robustness and ability to produce enzymes that maintain catalytic stability under extreme variations in pH and temperature—conditions inherent to disinfection and environmental management processes [6,7].

An example of a commercial product derived from *B. licheniformis* is the HPF blend (Prozyn^®^), which contains proteases that have demonstrated effective activity against parasites [5]. Thus, the in vitro reductions observed in this study in the groups treated with enzymes—either alone or in combination—reinforce evidence that proteolytic catalytic activity is critical to compromising oocyst integrity, thereby consolidating the potential of these macromolecules for research aimed at environmental control from a One Health perspective.

## 4. Materials and Methods

### 4.1. Oocyst Acquisition and Preparation

The oocysts were obtained from the feces of naturally infected animals at the Department of Veterinary Clinical Medicine and Surgery at the Federal University of Minas Gerais (UFMG), Brazil. Initially, the material was sieved to remove coarse particles and immersed in a 2.5% (*m*/*v*) potassium dichromate (K_2_Cr_2_O_7_) (Pró-químicos, lot. 05/0418, São Paulo, SP, Brazil) solution at a 1:2 ratio. To promote sporulation, the mixture was kept under adequate aeration for 7 to 10 days at 26 ± 1 °C. After this period, the suspension was stored under refrigeration at 4 ± 1 °C to preserve biological viability. Prior to the in vitro assays, the sample was purified by sequential centrifugation at 3600× *g* for 5 min in sterile distilled water to ensure complete removal of K_2_Cr_2_O_7_. The final step in oocyst concentration was performed using the Willis flotation method [42], yielding a clear suspension for the experiments.

The surface integrity and morphology of the oocysts were verified by optical microscopy, in accordance with the structural criteria established for the species [43]. The final concentration of the suspension was standardized to approximately 60 oocysts per 40 µL. All experimental procedures were conducted at the Laboratory of Applied Biotechnology and Biochemistry (LaBBA), Department of Chemistry (DQI), Federal University of Lavras (UFLA), located in Lavras, Minas Gerais, Brazil.

### 4.2. Obtaining the Enzymes

In this study, the individual and synergistic activities of two proteases from different biological sources were investigated. The first was a microbial protease derived from *B. licheniformis*, kindly donated by Prozyn^®^-Brazil as a purified blend (HPF). The second enzyme used was papain, a plant-derived protease (*C. papaya*), obtained commercially from (Dinâmica Química Contemporânea Ltd.a., Indaiatuba, São Paulo, Brazil) For the in vitro assays, the solutions were prepared at a concentration of 15% *w*/*v* by dissolving the enzymes in sterile distilled water (pH ~7.0) at room temperature (20–25 °C), ensuring complete solubilization and homogeneous distribution of the biocatalysts.

### 4.3. Enzyme Stability and Compatibility Assays

A biochemical compatibility and stability study was carried out to assess possible antagonistic or synergistic effects amongst proteases. The experimental protocol was structured in three treatments: (G1) a 15% (*w*/*v*) HPF microbial blend solution (Prozyn^®^, São Paulo, SP, Brazil), (G2) a 15% (*w*/*v*) vegetable papain solution, and (G3) an equal combination of HPF and papain, maintaining an individual concentration of 15% (*w*/*v*) for each enzyme. The treatments were packaged in sterile microtubes and incubated in a BOD chamber under controlled temperature (28 ± 1 °C) in the absence of light, mimicking the environment of biological tests. The enzymatic stability profile was evaluated at 0 h, 24 h, 48 h, and 72 h intervals. At intervals, the enzymatic stability profile was assessed. To measure the overall proteolytic activity, 100 μL aliquots were taken throughout each sampling time.

### 4.4. Proteolytic Activity

Proteolytic activity was measured using the caseinolytic method and the subsequent protocol: For 30 min, 100 µL of sample was incubated at 50 °C with 500 µL of 1.0% (*w*/*v*) casein (Êxodo Científica, lot. 2302150857, Sumaré, SP, Brazil) and 400 µL of 50 mM Tris-HCl buffer (pH 8.5) (Tris, Sigma, lot. BCBJ4922V, Barueri, SP, Brazil and HCl, Êxodo Científica, lot. 2206153888, Sumaré, SP, Brazil). After the reaction was stopped by adding 1.0 mL of 10.0% (*w*/*v*) trichloroacetic acid (TCA) (Synth, lot. 26076, Diadema, SP, Brazil), the activity was then measured. After centrifuging the suspensions for ten minutes at 10,000× *g*, the absorbance of the supernatant was measured at 280 nm. The quantity of enzyme that releases 1 µg of tyrosine per minute under the specified conditions is equal to one activity unit (U) [44].

### 4.5. In Vitro Experiments

To experiment, the groups were divided as follows: G1 (negative control—distilled H_2_O), G2 (positive control—0.04% *v*/*v* NaClO), G3 (15% papain treatment), G4 (15% HPF treatment), and G5 (concomitant treatment with papain and HPF, each at a 15% concentration). Each group consisted of seven replicates prepared in 200 µL microtubes; initially, each replicate received 40 µL of a clean *Cryptosporidium* spp. suspension containing approximately 60 oocysts. For groups G3 (15% papain treatment) and G4 (15% HPF treatment), 40 µL of the corresponding enzyme solution (15% *w*/*v*) was added. Finally, group G5 (concomitant papain and HPF treatment) received 20 µL of each enzyme, for a total of 40 µL of treatment. The microtubes were then capped and incubated for 72 h in a BOD incubator in the dark. After this period, the samples were transferred to microscope slides, and staining was performed according to the modified Ziehl-Neelsen method [45].

### 4.6. Preparation of Enzyme Structures (Ligands)

The three-dimensional structures of the proteases used in the experimental assays were retrieved from the RCSB Protein Data Bank (PDB) (https://www.rcsb.org/) [46]. For the plant protease, the X-ray diffraction crystal of papain from *C. papaya* was obtained under the PDB accession code 1PPP [47]. For the microbial protease, the crystal structure of Subtilisina Carlsberg from *B. licheniformis* was selected under the PDB code 1SCA [48]. Both files were submitted to a structural preparation and cleaning protocol using PyMOL software (v. 2.5, Educational-Use License), where all crystallographic water molecules (solvent), free organic heteroatoms (organic molecules), and interfering inorganic ions (inorganic ions) were removed [49]. The clean structures were saved as isolated monomers (Chain A) to represent the mature and functional monomeric unit of the enzymes, thereby eliminating steric noise from adjacent inactive chains, and rigid dynamic coordinates were established for the subsequent coupling stages.

### 4.7. Retrieval of Oocyst Wall Proteins (Receptors)

Due to the absence of experimentally resolved crystallographic structures for the structural proteins of the *Cryptosporidium* spp. oocyst wall in the Protein Data Bank (PDB) [46], the three-dimensional structures of the selected targets—including the structural matrix proteins COWP6 and COWP8, and the surface protein CpSP1—were retrieved directly from the AlphaFold Protein Structure Database (AFDB) (https://alphafold.ebi.ac.uk/) [23,26]. Initially, their respective gene accession codes (cgd4_3090, cgd6_200, and cgd7_5140) were located in the National Center for Biotechnology Information (NCBI) (https://www.ncbi.nlm.nih.gov) database and cross-referenced with the UniProt database (https://uniprot.org) to map their primary accession IDs [50]. Using these UniProt IDs, the pre-calculated, highly reliable 3D models generated by the open-source AlphaFold 2 algorithm were downloaded [12,51,52]. The generated files were exported in PDB format and inspected for local quality scores (predicted Local Distance Difference Test—pLDDT) before molecular docking.

### 4.8. Protein–Protein Molecular Docking Simulations

Protein–protein molecular docking simulations were performed using the HDOCK web server (http://hdock.phys.hust.edu.cn/) [53] to investigate predicted binding scores and binding mechanisms between the cleaving enzymes (Papain and Subtilisin) and the structural proteins of the *C. parvum* oocyst wall. Given the rigid nature of the oocyst wall matrix and the fact that protease-target interactions are transient, HDOCK was chosen to perform an unbiased global surface scan (blind docking) without active-site restrictions, applying its default sampling parameters for an exhaustive rigid-body search and knowledge-based scoring. While deep-learning complex predictors excel at modeling stable multimeric assemblies, rigid-body docking remains a robust approach to evaluate transient enzymatic poses based on shape complementarity without the bias of co-folding. Furthermore, because the intrinsic disorder predicted in monomeric COWPs is highly restricted in vivo due to extensive cross-linking [23], rigid docking directed at their ordered domains (pLDDT > 70) is a biologically sound approximation for predicting favorable binding interfaces. The final generated conformations were ranked based on the estimated binding free energy (ΔG, in kcal·mol^−1^) and dissociation constant (Kd, in molar units), which were estimated using the PRODIGY server (https://wenmr.science.uu.nl/prodigy/) [32,54]. These values were used as primary indicators of static binding affinity, rather than direct measures of catalytic cleavage. In addition, intermolecular contacts (ICs) were quantified and classified into charged–charged, charged–polar, charged–apolar, polar–polar, polar–apolar, and apolar–apolar interactions. These parameters provided a detailed profile of the binding interface, enabling the identification of complexes with both strong predicted affinity and reliable structural confidence.

Specific intermolecular interactions in the enzyme–receptor complexes of best affinity were mapped and analyzed using PyMOL [49]. Graphical inspection included highlighting the binding interface and zooming into the contact region to facilitate comparative analysis of the docking results, focusing strictly on spatial fit and structural reliability.

### 4.9. Structural Alignment and Confidence Assessment

Docking-derived complexes were aligned to the original receptor chain using PyMOL [49] to ensure structural consistency. Protein–protein interfaces for graphical analysis were defined as residues within 4 Å of the ligand chain [32]. The number of amino acids contributing to each interface was determined by counting carbon alpha (Cα) atoms. To ensure accurate representation of structural reliability, the predicted COWP receptors were colored according to the standard AlphaFold 2 pLDDT confidence scheme: very high confidence (pLDDT > 90, blue), confident (90 > pLDDT > 70, teal), low confidence (70 > pLDDT > 50, yellow), and very low confidence (pLDDT < 50, orange) [51]. Because the proteases (1SCA and 1PPP) are experimentally determined crystallographic structures retrieved from the PDB, pLDDT scores are not applicable; therefore, the enzyme chains were rendered in a fixed contrasting color (magenta). For graphical documentation, the interfaces were further inspected with local zoom and translucent surface representation. Specific intermolecular interactions, such as hydrogen bonds, were deliberately excluded from the graphical rendering to avoid visual clutter and overinterpretation without direct experimental residue-level validation.

### 4.10. Statistical Analysis

The experimental data, including the enzymatic activity (U/mL) across different incubation times and the number of intact *Cryptosporidium* spp. oocysts obtained for each treatment, were first evaluated for normality. Subsequently, statistical comparisons between groups were performed using One-Way Analysis of Variance (ANOVA) followed by Student’s *t*-tests for pairwise inferences, assessing significance levels of 1% and 5% (*p* < 0.01 and *p* < 0.05). All statistical analyses were executed using the BioEstat version 5.0 statistical package. [55]. The mean mortality percentage was calculated based on the number of intact oocysts counted, using the following Equation (1) [56]:

(1) $$\%\;\mathrm{Mortality}\;=\frac{\left(\mathrm{Average}\;\mathrm{number}\;\mathrm{of}\;\mathrm{control}\;\mathrm{oocists}\;-\;\mathrm{Average}\;\mathrm{number}\;\mathrm{of}\;\mathrm{treated}\;\mathrm{oocists}\right)\;\times \;100}{\left(\mathrm{Average}\;\mathrm{number}\;\mathrm{of}\;\mathrm{control}\;\mathrm{oocists}\right)}$$

## 5. Conclusions

This study shows that protease-mediated biochemical control has great potential to serve as an effective and sustainable alternative for the destruction of *Cryptosporidium* spp. oocysts. In this in vitro study, the microbial protease (*B. licheniformis*) exhibited efficacy statistically equivalent to that of conventional control with sodium hypochlorite, while papain showed inferior but still significant performance. The structural damage and reductions observed suggest the hydrolysis of the oocyst’s external and internal proteins. Additionally, the in silico findings complemented the results, revealing that Carlsberg subtilisin exhibits more favorable docking scores and a greater molecular binding cleft than papain, denoting a higher binding potential toward the major structural proteins of the oocyst matrix. However, further kinetic assays are required to confirm the precise biochemical pathways of peptide bond cleavage.

Thus, this study demonstrated that the use of proteases for the control of *Cryptosporidium* spp. oocysts has the potential to serve as a control alternative aimed at reducing dependence on toxic chemical disinfectants, mitigating the formation of harmful environmental byproducts, and is important within the framework of One Health, with high potential for integration into water sanitation protocols and veterinary management. Future research directions must include in vivo infectivity assays (using animal models) to definitively confirm the loss of oocyst viability, enzyme optimization studies (such as adjusting pH and temperature via Response Surface Methodology), and field-scale validation in complex environmental matrices to translate this biotechnological potential into practical use.

## Figures and Tables

**Table 1:** Presentation of protein–protein docking models and parameters for evaluating the structural reliability and predicted binding scores of enzymatic interactions with the oocyst wall. The values for estimated binding free energy (ΔG, kcal·mol^−1^), dissociation constant (Kd, mol·L^−1^), and predicted intermolecular contact (IC) profiles—including charged, polar, and hydrophobic interactions—are shown. Results obtained for the structural proteins of *Cryptosporidium* spp. (COWP6, COWP8, and CpSP1) and the protease papain (1PPP).

Protein (Receptor)	Docking Model for Papain (1PPP)	ΔG (kcal/mol)	Kd (M)	Interatomic Contact (IC) Profile
COWP6	Model 3	−12.7	5.2 × 10^−10^	Predominantly hydrophobic (polar-nonpolar + nonpolar-nonpolar)
COWP8	Model 10	−13.5	1.3 × 10^−10^	Strongly hydrophobic (polar-nonpolar + nonpolar-nonpolar)
CpSP1	Model 9	−10.7	1.4 × 10^−8^	Small interface, moderately hydrophobic

**Table 2:** Presentation of protein–protein docking models and parameters for evaluating the structural reliability and predicted binding scores of enzymatic interactions with the oocyst wall. The values for estimated binding free energy (ΔG, kcal·mol^−1^), dissociation constant (Kd, mol·L^−1^), and predicted intermolecular contact (IC) profiles—including charged, polar, and hydrophobic interactions—are shown. Results obtained for the structural proteins of *Cryptosporidium* spp. (COWP6, COWP8, and CpSP1) and the Carlsberg subtilisin protease (1SCA).

Protein (Receptor)	Docking Model for Subtilisin Carlsberg (1SCA)	ΔG (kcal/mol)	Kd (M)	Interatomic Contact (IC) Profile
COWP6	Model 3	−13.3	1.7 × 10^−10^	Predominantly hydrophobic (polar-nonpolar + nonpolar-nonpolar)
COWP8	Model 6	−15.2	6.9 × 10^−12^	Strongly hydrophobic (polar-nonpolar + nonpolar-nonpolar)
CpSP1	Model 4	−12.7	5.2 × 10^−10^	Small interface, moderately hydrophobic

## Data Availability

The original contributions presented in this study are included in the article/Supplementary Materials. Further inquiries can be directed to the corresponding author.

## Supplementary Materials

The following supporting information can be downloaded at: https://www.mdpi.com/article/10.3390/molecules31162919/s1, Table S1. Interfacial residue mapping of the predicted binding interfaces between *Cryptosporidium* spp. structural proteins (COWP6, COWP8, CpSP1) and the proteases papain (1PPP) and subtilisin Carlsberg (1SCA). Interacting residues were defined by a heavy-atom distance cutoff of ≤4.0 Å. Asterisks indicate structural relevance: (*) denotes protease active site/catalytic triad residues; (**) denotes potential cleavage targets (Cysteine and Tyrosine residues) on the oocyst wall proteins.

## References

[1] Farhan M., Hasani I.W., Khafaga D.S., Ragab W.M., Ahmed Kazi R.N., Aatif M., Muteeb G., Fahim Y.A. (2025). Enzymes as catalysts in industrial biocatalysis: Advances in engineering, applications, and sustainable integration. Catalysts.

[2] De Freitas Soares F.E., Ferreira J.M., Genier H.L.A., Al-Ani L.K.T., Aguilar-Marcelino L. (2023). Biological control 2.0: Use of nematophagous fungi enzymes for nematode control. J. Nat. Pestic. Res..

[3] Sousa S.F., Ramos M.J., Lim C., Fernandes P.A. (2015). Relationship between enzyme/substrate properties and enzyme efficiency in hydrolases. ACS Catal..

[4] Ferreira C.B., Mamani R.C.C., Sousa M.R., Braga F.R., Moreira T.F., Bianchi M.L., Soares F.E.F. (2024). *In vitro* evaluation of the anthelmintic potential of proteases from garlic peel (*Allium sativum*). Contrib. Cienc. Soc..

[5] Silva A.C., de Souza D.C., da Silva A.T., Mamani R.C.C., Moreira T.F., Braga F.R., Soares F.E.F. (2025). *In vitro* evaluation of *Bacillus licheniformis* protease on the nematodes *Panagrellus* sp., *Meloidogyne incognita, Haemonchus* spp. and *Trichostrongylus* spp.. Next Res..

[6] Muras A., Romero M., Mayer C., Otero A. (2021). Biotechnological applications of *Bacillus Licheniformis*. Crit. Rev. Biotechnol..

[7] He H., Yu Q., Ding Z., Zhang L., Shi G., Li Y. (2023). Biotechnological and food synthetic biology potential of platform strain: *Bacillus licheniformis*. Synth. Syst. Biotechnol..

[8] Ward O.P. (2019). Proteases. Compr. Biotechnol..

[9] Angulo M., Márquez M.C. (2023). A green technology approach using enzymatic hydrolysis to valorize meat waste as a way to achieve a circular economy. Appl. Sci..

[10] De Souza D.C., Gomes E.H., Puentes L.B.F., Soares D.S., Da Silva D.V., Albuquerque L.B., Dos Santos P.H.D., Facury T.M., Braga F.R., De Freitas Soares F.E. (2024). *Duddingtonia flagrans* and its crude proteolytic extract: Concomitant action in sheep coprocultures. Vet. Res. Commun..

[11] Innes E.A., Chalmers R.M., Wells B., Pawlowic M.C. (2020). A One Health approach to tackle cryptosporidiosis. Trends Parasitol..

[12] Rideout H., Cook A.J., Whetton A.D. (2024). Understanding the *Cryptosporidium* species and their challenges to animal health and livestock species for informed development of new, specific treatment strategies. Front. Parasitol..

[13] Bamaiyi P.H., Redhuan N.E.M. (2016). Prevalence and risk factors for cryptosporidiosis: A global, emerging, neglected zoonosis. Asian Biomed..

[14] Widodo W.T., Khairullah A.R., Pratama B.P., Ambarika R., Furqoni A.H., Kristianto S., Wardhani B.W.K., Widoretno W., Khariri K., Hermawati L. (2025). Cryptosporidiosis: A global threat to human and animal health. Open Vet. J..

[15] Mateo M., de Mingo M.H., de Lucio A., Morales L., Balseiro A., Espí A., Carmena D. (2017). Occurrence and molecular genotyping of *Giardia duodenalis* and *Cryptosporidium* spp. in wild mesocarnivores in Spain. Vet. Parasitol..

[16] Veronesi F., Deak G., Diakou A. (2023). Wild mesocarnivores as reservoirs of endoparasites causing important zoonoses and emerging bridging infections across Europe. Pathogens.

[17] Fayer R., Ungar B.L. (1986). *Cryptosporidium* spp. and cryptosporidiosis. Microbiol. Rev..

[18] Helmy Y.A., Hafez H.M. (2022). Cryptosporidiosis: From Prevention to Treatment, a Narrative Review. Microorganisms.

[19] Kotloff K.L., Nataro J.P., Blackwelder W.C., Nasrin D., Farag T.H., Panchalingam S., Wu Y., Sow S.O., Sur D., Breiman R.F. (2013). Burden and aetiology of diarrhoeal disease in infants and young children in developing countries (the Global Enteric Multicenter Study, GEMS): A prospective, case-control study. Lancet.

[20] Ras R., Abdelkhalek A., Raya-Álvarez E., Shady R.M., AlGabbani Q., Abdelbaset A.E., Saleem A.S.A., Shukry M., Agil A., Elmahallawy E.K. (2026). *Cryptosporidium* within a One health framework: A comprehensive review of public health impact, environmental concerns, and emerging strategies for prevention and treatment. Front. Vet. Sci..

[21] Bulumulla S., Xiao L., Feng Y., Ash A., Aleri J., Ryan U., Barbosa A.D. (2025). *Cryptosporidium* in cattle: Assessing the zoonotic risk. Curr. Res. Parasitol. Vector-Borne Dis..

[22] Golomazou E., Mamedova S., Eslahi A.V., Karanis P. (2024). *Cryptosporidium* and agriculture: A review. Sci. Total Environ..

[23] Bacchetti R., Stevens S., Lemgruber L., Gonzalez Oliva M.A., Sands E.M., Alexandrou K., Tinti M., Robinson L., Hanna J.C., Seizova S. (2025). *Cryptosporidium* oocyst wall proteins are true components of the oocyst wall and COWP8 is not required for parasite transmission. PLoS Pathog..

[24] Chatterjee A., Banerjee S., Steffen M., O’Connor R.M., Ward H.D., Robbins P.W., Samuelson J. (2010). Evidence for mucin-like glycoproteins that tether sporozoites of *Cryptosporidium parvum* to the inner surface of the oocyst wall. Eukaryot. Cell.

[25] Harris J.R., Petry F. (1999). *Cryptosporidium parvum*: Structural components of the oocyst wall. J. Parasitol..

[26] Okasha H., El-Kalamawy H.A., Mashaal A.R., El-Wakil E.S. (2026). Development and validation of qPCR assay targeting COWP conserved region for sensitive detection and quantification of *Cryptosporidium* infections. Vet. Parasitol..

[27] Dąbrowska J., Sroka J., Cencek T. (2023). Investigating *Cryptosporidium* spp. Using Genomic, Proteomic and Transcriptomic Techniques: Current Progress and Future Directions. Int. J. Mol. Sci..

[28] Wang L., Wang Y., Cui Z., Li D., Li X., Zhang S., Zhang L. (2022). Enrichment and proteomic identification of *Cryptosporidium parvum* oocyst wall. Parasites Vectors.

[29] Bell E.L., Finnigan W., France S.P., Green A.P., Hayes M.A., Hepworth L.J., Lovelock S.L., Niikura H., Osuna S., Romero E. (2021). Biocatalysis. Nat. Rev. Methods Primers.

[30] Murphy D.L. (2024). The Application of Advanced Structural Bioinformatics Methods to Parasitic Membrane Proteins. Doctoral Dissertation.

[31] Xu C., He Q., Zhu Z., Li K. (2025). Propolis improves intestinal barrier function against *Cryptosporidium parvum* via NLRP6 inflammasome. mBio.

[32] Vangone A., Bonvin A.M.J.J. (2015). Contact-based prediction of binding affinity in protein-protein complexes. eLife.

[33] United Nations Transforming Our World: The 2030 Agenda for Sustainable Development. Resolution Adopted by the General Assembly 2015, A/RES/70/1, 2015. https://docs.un.org/en/A/RES/70/1.

[34] Souza D.C., Figueroa L.B.P., Silva A.T., Ferreira D.P., Ferraz C.M., Braga F.R., Soares F.E.F. (2025). *In vitro* Biochemical Control of *Taenia solium* and *Taenia saginata* Eggs. Acta Parasitol..

[35] Adeyemo F.E., Singh G., Reddy P., Bux F., Stenström T.A. (2019). Efficiency of chlorine and UV in the inactivation of *Cryptosporidium* and *Giardia* in wastewater. PLoS ONE.

[36] Javed K., Alkheraije K.A. (2023). Cryptosporidiosis: A foodborne zoonotic disease of farm animals and humans. Pak. Vet. J..

[37] Caffrey C.R., Goupil L., Rebello K.M., Dalton J.P., Smith D. (2018). Cysteine proteases as digestive enzymes in parasitic helminths. PLoS Neglected Trop. Dis..

[38] Siqueira L.N., de Souza D.C.T., Mamani R.C.C., Figueroa L.B.P., Albuquerque L.B., Moreira T.F., Braga F.R., Soares F.E.F. (2025). *In vitro* Action of Papaya (*Carica Papaya*) Latex and Pure Papain Against *Eimeria Bovis* Oocysts. Acta Parasitol..

[39] Wang R., Qi M., Jingjing Z., Sun D., Ning C., Zhao J., Zhang L., Xiao L. (2011). Prevalence of *Cryptosporidium baileyi* in ostriches (Struthio camelus) in Zhengzhou, China. Vet. Parasitol..

[40] Wood M., Simmonds L., MacAdam J., Hassard F., Jarvis P., Chalmers R.M. (2019). Role of filtration in managing the risk from *Cryptosporidium* in commercial swimming pools–a review. J. Water Health.

[41] Berger J., Asenjo C.F. (1940). Anthelmintic activity of crystalline papain. Science.

[42] Willis H.H.A. (1921). A simple levitation method for the detection of hookworm ova. Med. J. Aust..

[43] Santin M. (2020). *Cryptosporidium* and *Giardia* in Ruminants. Vet. Clin. N. Am. Food Anim. Pract..

[44] Braga F.R., Araujo J.M., Tavela A.D.O., Araújo J.V.D., Soares F.E.D.F., Geniêr H.L.A., Lima W.D.S., Mozzer L.R., Queiroz J.H.D. (2011). Atividade larvicida do extrato bruto enzimático do fungo *Duddingtonia flagrans* sobre larvas de primeiro estádio de *Angiostrongylus vasorum*. Rev. Soc. Bras. Med. Trop..

[45] Henriksen S.A., Pohlenz J.F. (1981). Staining of Cryptosporidia by a Modified Ziehl-Neelsen Technique. Acta Vet. Scand..

[46] Berman H.M., Westbrook J., Feng Z., Gilliland G., Bhat T.N., Weissig H., Shindyalov I.N., Bourne P.E. (2000). The Protein Data Bank. Nucleic Acids Res..

[47] Kim M.J., Yamamoto D., Matsumoto K., Inoue M., Ishida T., Mizuno H., Sumiya S., Kitamura K. (1992). Crystal structure of papain-E64-c complex. Binding diversity of E64-c to papain S2 and S3 subsites. Biochem. J..

[48] Fitzpatrick P.A., Steinmetz A.C.U., Ringe D., Klibanov A.M. (1993). Enzyme crystal structure in a neat organic solvent. Proc. Natl. Acad. Sci. USA.

[49] Schrödinger L.L.C. (2021). The PyMOL Molecular Graphics System.

[50] Sayers E.W., Beck J., Bolton E.E., Bourexis D., Brister J.R., Canese K., Comeau D.C., Funk K., Kim S., Klimke W. (2021). Database resources of the national center for biotechnology information. Nucleic Acids Res..

[51] Jumper J., Evans R., Pritzel A., Green T., Figurnov M., Ronneberger O., Tunyasuvunakool K., Bates R., Žídek A., Potapenko A. (2021). Highly accurate protein structure prediction with AlphaFold. Nature.

[52] Mattice E.B. (2024). Drug Target Identification Methods for Cryptosporidium.

[53] Yan Y., Zhang D., Zhou P., Li B., Huang S.Y. (2017). HDOCK: A web server for protein–protein and protein–DNA/RNA docking based on a hybrid strategy. Nucleic Acids Res..

[54] Xue L., Rodrigues J., Kastritis P., Bonvin A.M.J.J., Vangone A. (2016). PRODIGY: A web-server for predicting the binding affinity in protein-protein complexes. Bioinformatics.

[55] Ayres M., Ayres J.R., Ayres D.L., Santos A.S. (2003). BioEstat 3.0: Aplicações Estatísticas nas Áreas das Ciências Biológicas e Médicas.

[56] Mendoza-De Gives P., Vazquez-Prats V.M. (1994). Reduction of *Haemonchus contortus* infective larvae by three nematophagous fungi in sheep faecal cultures. Vet. Parasitol..

