# Supplementary material for: Enzymatic Degradation of Cryptosporidium spp. Oocysts: A Combined In Silico and In Vitro Study
Source: Molecules. 2026 Aug 21;31(16):2919. doi: 10.3390/molecules31162919 (PMC13515647; doi:10.3390/molecules31162919)
Supplement: Supplementary file 1 [file molecules-31-02919-s001.zip › molecules-4442778-supplementary.pdf]

Supplementary Table S1. Interfacial residue mapping of the predicted binding interfaces between *Cryptosporidium* spp. structural proteins (COWP6, COWP8, CpSP1) and the proteases papain (1PPP) and subtilisin Carlsberg (1SCA). Interacting residues were defined by a heavy-atom distance cutoff of ≤ 4.0 Å. Asterisks indicate structural relevance: (*) denotes protease active site/catalytic triad residues; (**) denotes potential cleavage targets (Cysteine and Tyrosine residues) on the oocyst wall proteins.

Predicted Complex	Estimated ΔG (kcal/mol)	Protease Interacting Residues (Chain B)	Target Protein Interacting Residues (Chain A)
COWP6 - 1PPP	-12.7	Gly-66, Gly-109, Val-110, Arg-111, Lys-156, Val-157	Pro-82, Pro-477, Val-481, His-484, Gly-485, Val-487, Leu-488, Pro-490, Asp-491
COWP6 - 1SCA	-13.3	Gly-157, Ser-188, Phe-189, Thr-211, Thr-213, Ala-215, Thr-216, Leu-217	Glu-499, Lys-502, Ser-504, Ser-506, His-512, Phe-513
COWP8 - 1SCA	-15.2	Ala-37, Ser-38, Pro-40, Asn-43, Val-44, Val-45, Gly-53, Ser-87, Lys-237	Met-142, Leu-155, Gln-156, Lys-157, Ser-158, Val-159, Ser-160, Gly-247, Phe-248, Gly-249, Asn-264
COWP8 - 1PPP	-13.5	Gly-109, Val-110, Arg-111, Val-133, Lys-156, Val-157, Asp-158	Asp-125, Ala-263, Gly-265, Val-279, Pro-371
CpSP1 - 1PPP	-10.7	Asp-57, Ser-205	Lys-104, Leu-105, Val-108, Thr-109, Lys-133
CpSP1 - 1SCA	-12.7	Asn-158, Gly-160, Ser-161, Thr-162, Ala-194, Gly-258, Ser-259, Phe-261	Ile-107, Val-108, Thr-109, Ile-135, **Tyr-137** **
